# A New App for At-Home Cognitive Training: Description and Pilot Testing on Patients with Multiple Sclerosis

**DOI:** 10.2196/mhealth.4269

**Published:** 2015-08-31

**Authors:** Andrea Tacchino, Ludovico Pedullà, Laura Bonzano, Claudio Vassallo, Mario Alberto Battaglia, Gianluigi Mancardi, Marco Bove, Giampaolo Brichetto

**Affiliations:** ^1^Italian Multiple Sclerosis FoundationScientific Research AreaGenoaItaly; ^2^Department of Experimental Medicine, Section of Human PhysiologyUniversity of GenoaGenoaItaly; ^3^Department of Neuroscience, Rehabilitation, Ophthalmology, Genetics, Maternal and Child HealthUniversity of GenoaGenoaItaly; ^4^Magnetic Resonance Research Centre on Nervous System DiseasesUniversity of GenoaGenoaItaly; ^5^Department of Life ScienceUniversity of SienaItaly

**Keywords:** tablet, mobile phone, mobile device, cognitive rehabilitation, cognitive impairment, working memory, self-management, adaptive working load algorithms, usability

## Abstract

**Background:**

Cognitive impairment is common in people with neurological diseases and severely affects their social and professional life. It has been shown that intensive and personalized cognitive rehabilitation (CR), based on working memory exercises, leads to improved cognitive status of healthy and cognitive-impaired subjects. New technologies would help to promote accessible, at-home, and self-managed CR interventions.

**Objective:**

The aim of this paper is to describe the design of Cognitive Training Kit (COGNI-TRAcK), an app for mobile devices, to self-administer an at-home, intensive, and personalized CR intervention based on working memory exercises, and test its disposability-to-use (usability, motivation to use, compliance to treatment) on cognitive-impaired patients with multiple sclerosis (MS).

**Methods:**

COGNI-TRAcK includes user-friendly interfaces for personal data input and management and for CR intervention configurations. Inner routines automatically implement adaptive working load algorithms and allow data processing and analysis. A dedicated team developed COGNI-TRAcK with C# programming language, by using the platform Xamarin Studio 4.0.10 for Android (API level 15 and following). Three exercises based on working memory are now available. To assess the disposability-to-use of the system, patients with MS were selected as likely users due to the young age of disease onset. Cognitive-impaired patients with MS (N=16) with a mean age of 49.06 years (SD 9.10) and a mean score of 3.75 (SD 1.92) on the Expanded Disability Status Scale (EDSS) were submitted to an 8-week at-home intervention administered by the app. The intervention consisted of 5 daily scheduled 30-minute sessions per week. Disposability-to-use of COGNI-TRAcK was investigated by means of a questionnaire administered to patients at the end of the training.

**Results:**

The adherence to the treatment was 84% (33.4/40). Of the patients with MS, 94% (15/16) understood the instructions given, 100% (16/16) felt independent to use COGNI-TRAcK at home, 75% (12/16) found the exercises interesting, and 81% (13/16) found the exercises useful and were motivated to use the app again. Moreover, during the exercises, patients with MS were highly motivated to perform well (mean score 3.19/4, SE 0.16), experienced rather low levels of stress (mean score 2.19/4, SE 0.26), were not bored (mean score 1.81/4, SE 0.30), and felt amusement (mean score 2.25/4, SE 0.23).

**Conclusions:**

As COGNI-TRAcK is highly usable, motivating, and well-accepted by patients with MS, its effectiveness can now be investigated. To improve COGNI-TRAcK, new releases should contain more working memory exercises, have enhanced perceived amusement, and promote Internet communication procedures for data transfer and fostering remote control of the intervention.

## Introduction

Cognitive impairment is common in people with neurological diseases [1,2] and it can deleteriously impact their occupational profile, social participation, and quality of life [3,4]. The alleviation of deficits on cognitive functioning is the main goal of cognitive rehabilitation (CR), and research should address the best way to administer CR to patients.

Recently, many studies on CR demonstrated that a training based on working memory produced relevant positive effects on cognitive status [5]. Working memory is defined as a limited capacity storage system involved in maintenance and manipulation of information over short periods of time, and it is involved in the execution of higher-order daily cognitive activities (ie, reading, learning, and mental calculation) [6]. Working memory capacity has been traditionally thought to be constant in healthy adults, although differences were observed depending on education level, age, and potential deficits in cognitive status. Actually, some recent studies demonstrated that a specific training of working memory not only produced an improvement in specifically trained aspects but a more general gain of working memory [6-8]. Furthermore, a training of working memory seems to positively influence a wide range of cognitive functions. In particular, a transfer effect was found on attention [9,10], cognitive inhibition [11], non-verbal reasoning [12], reading [11], and arithmetic [13,14]. In a review conducted by Takeuchi et al [5], the authors stressed the importance of several factors that may affect training efficacy. Interestingly, besides task types, subject motivation and arousal status, an adaptive working load (adjusting the task difficulty to the subjects' performance), and the intensiveness of training (quantity of training per day per week) were mentioned as crucial features of working memory training [12,15,16].

It has been clearly shown that an adaptive and intensive working memory training produces significant changes in healthy subjects' brain structure (white and gray matter) associated with an improvement in cognitive functions [16,17]. In addition, working memory impairments can also limit and/or restrict participation in daily activities [18].

In an era of inadequate economic resources to invest in health care, it is not possible to guarantee a constant and intensive outpatient administration of CR, seriously limiting the effects of the interventions. Indeed, proper CR programs based on traditional tools (eg, paper and pencil) require qualified professionals, outpatient facilities, and intensive treatment sessions which often lead to economic burdens at the expense of the user or the health care system. In this context, it would be desirable to promote a new CR management system in which clients, remotely supervised and assisted by their team through procedures based on an Internet network, can self-administer at-home rehabilitation programs. Computerized systems may be particularly suitable to attain this aim by guaranteeing very efficient and user-friendly CR tools that could be used at-home by patients either independently (eg, at early stages of neurological disease), confined to their home by disease progression, or other clinical constraints. In particular, computer-based CR tools usually implemented on laptops and/or desktop computers, have been shown to be effective in improving cognitive functions in patients with stroke [19], Alzheimer's disease [20], or multiple sclerosis (MS) [21], confirming that they could be considered an excellent option for CR. With recent technological innovations, researchers are challenged to quickly develop cognitive treatment programs for mobile phones and tablets, thus making home-based CR exercise management and the (self)-administration of intensive CR interventions more feasible to patients and their caregivers. However, particular attention must be taken when implementing algorithms for automatic working load adaptation and automatic procedures for intensiveness regulation that most current computer-based CR tools do not incorporate.

The aim of the present study is to describe Cognitive Training Kit (COGNI-TRAcK), a mobile phone and tablet-based app for the home, or self-administration, of an actual intensive CR intervention based on working memory exercises. The main feature of COGNI-TRAcK is the implementation of automatic adaptive working load algorithms and procedures for intensiveness regulation.

A pilot test on patients with MS was performed to evaluate the adherence to a self-administered CR intervention and its disposability-to-use (usability, motivation to use, compliance to treatment). Patients with MS were chosen as a testing population for two main reasons. First, since approximately 40-65% of patients with MS show disabling cognitive impairments [22] involving memory, complex attention, information processing speed, executive functions, and visuospatial abilities [2], systems for intensive cognitive interventions are required. Second, MS affects patients of all ages making them the ideal end-users of our system. Indeed, studies showed that most of patients with MS are <65 years old [23], an age group consisting of digital natives and digital immigrants and considered the most probable technology consumers [24].

In fact, the evaluation of adherence and disposability-to-use is fundamental to propose a future, large-sample study that examines the effectiveness of a CR intervention with COGNI-TRAcK on patients with MS using specific clinical outcomes.

## Methods

### Overview

COGNI-TRAcK allows patients to have access to tailored CR interventions at any time of the day, thus increasing adherence to the scheduled rehabilitative treatment. In order to assess disposability-to-use, a tablet version of the COGNI-TRAcK app was tested on cognitively-impaired patients with MS recruited at the Italian Multiple Sclerosis Society (AISM) Rehabilitation Centre of Genoa (Italy). Ethical approval for the pilot testing was obtained from the Ethics Committee of Azienda Ospedaliera San Martino, Genoa, Italy, in 2011.

### Development of the COGNI-TRAcK App

#### Overview

The development team first met in January, 2012 to discuss the project plan. An information technology (IT) specialist wrote the software program for COGNI-TRAcK with C# programming language using the platform Xamarin Studio 4.0.10, the version customized to create apps for the Android operating system (version 4.0, API level 15 and following). The development team met on a bi-weekly basis to resolve issues and review the progress of the software. A stakeholder team composed of the development team, a psychologist, and 3 patients (not included in the pilot testing) met 4 times during the development of COGNI-TRAcK and considered the final version suitable for patients with MS.

COGNI-TRAcK includes the following 4 main parts (1) the graphic user interface (GUI) allowing the administrator (eg, the clinician) to add personal data of new patients, to retrieve and to manage them, to set parameters of the working memory exercises, and to select general options of configuration (Multimedia Appendices 1-4), (2) the database, (3) routines for data processing, and (4) routines implementing adaptive working load algorithms. COGNI-TRAcK was conceived and designed with a modular structure able to evolve by adding new modules, such as exercises for working memory or other cognitive rehabilitative functions and electronic forms for compiling standardized scales for clinical evaluation.

Although not validated here as this study focused on disposability-to-use, the app was designed with a feature allowing for Internet-based data transfer between the device and a server installed at the rehabilitation unit for remote monitoring and management by the clinical team.

#### Working Memory Exercises Section

##### Overview

The working memory exercises section enables access to the editing of several exercise types. For each exercise type, records can be created and saved into the database, each one containing a specific configuration of parameters.

During the treatment protocol setting, the administrator directs the patient to the appropriate records in the database. Each record can be assigned more than once and each assignment corresponds to a trial that the patient will have to execute. In this modality of assignment, the administrator must set the complete protocol treatment in advance. This modality, however, does not use adaptive working load algorithms and is not preferred because the effectiveness of the rehabilitation intervention cannot be guaranteed. Therefore, the preferred way to set up a rehabilitation intervention is to make use of adaptive working load algorithms where the administrator has to assign only the starting record for each exercise type, corresponding to the first trial that the patient will perform. At the end of the first trial, a new record is automatically generated into the database and then assigned to the patient. This new record contains a new configuration of parameters calculated on the output of the applied adaptive working load algorithm. Increments or decrements in parameter values depend on the performance of the patient. The automatically generated record results are linked specifically to the patient and will not be assigned to another subject. To date, COGNI-TRAcK implements the following 3 types of working memory exercises.

##### Visuospatial Working Memory

**Figure 1:**
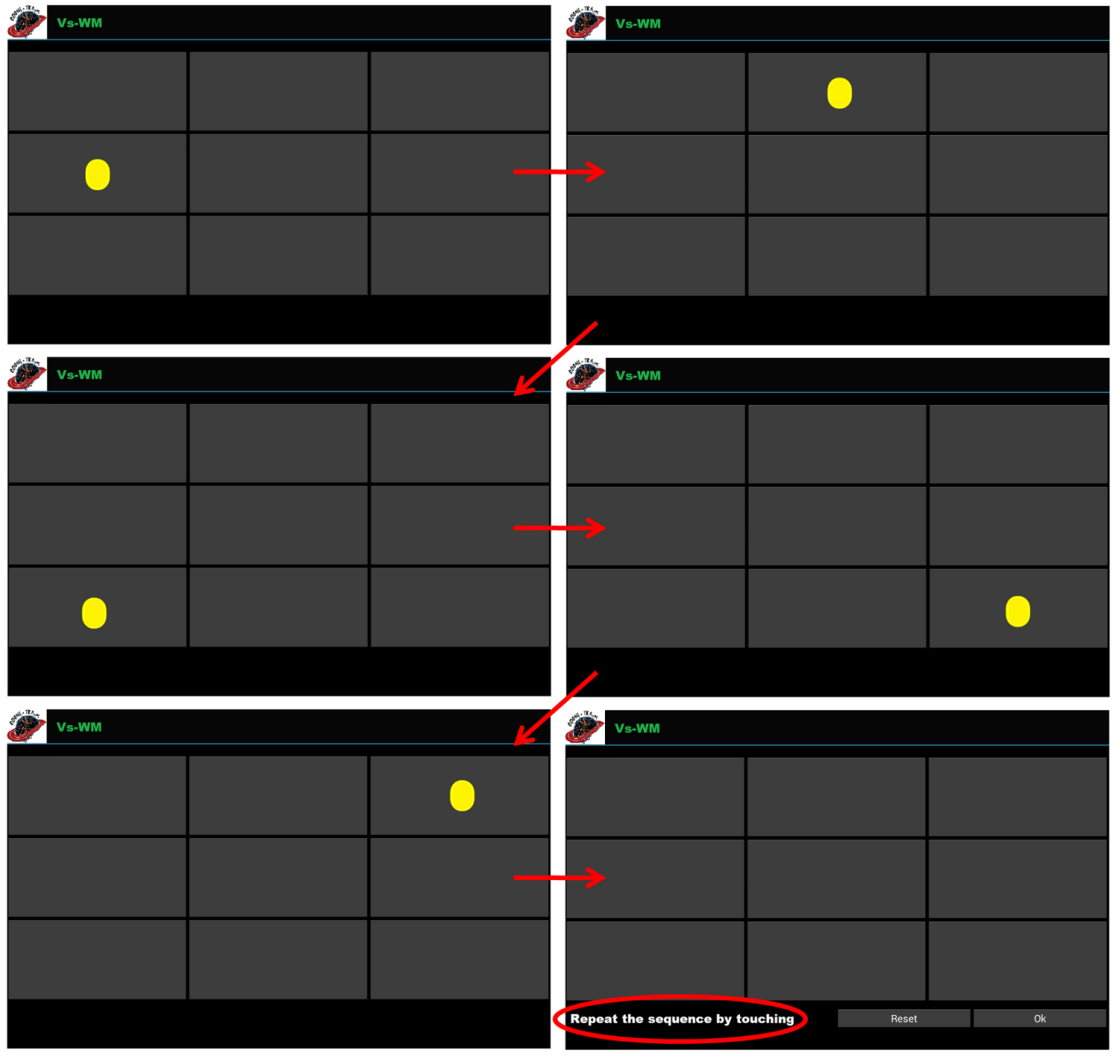
An example of visuospatial working memory (Vs-WM) exercises in which patients have to remember a random sequence of 5 circles consecutively presented (the temporal order is defined following the direction of the arrows). Task-specific parameters for Vs-WM include grid size (minimum 2×2; maximum not defined), number of stimuli composing the sequence (minimum 1; maximum not defined), and the rate of stimuli presentation. Adaptive working load algorithms could operate on all the task-specific parameters to increment the difficulty level.

In this type, patients have to remember a random sequence of visual stimuli (colored circles) presented one at a time in a grid-like interface; after the last stimulus presentation, they are asked to correctly reproduce the sequence by touching the corresponding locations on the device screen (Figure 1).

##### Operation N-Back

**Figure 2:**
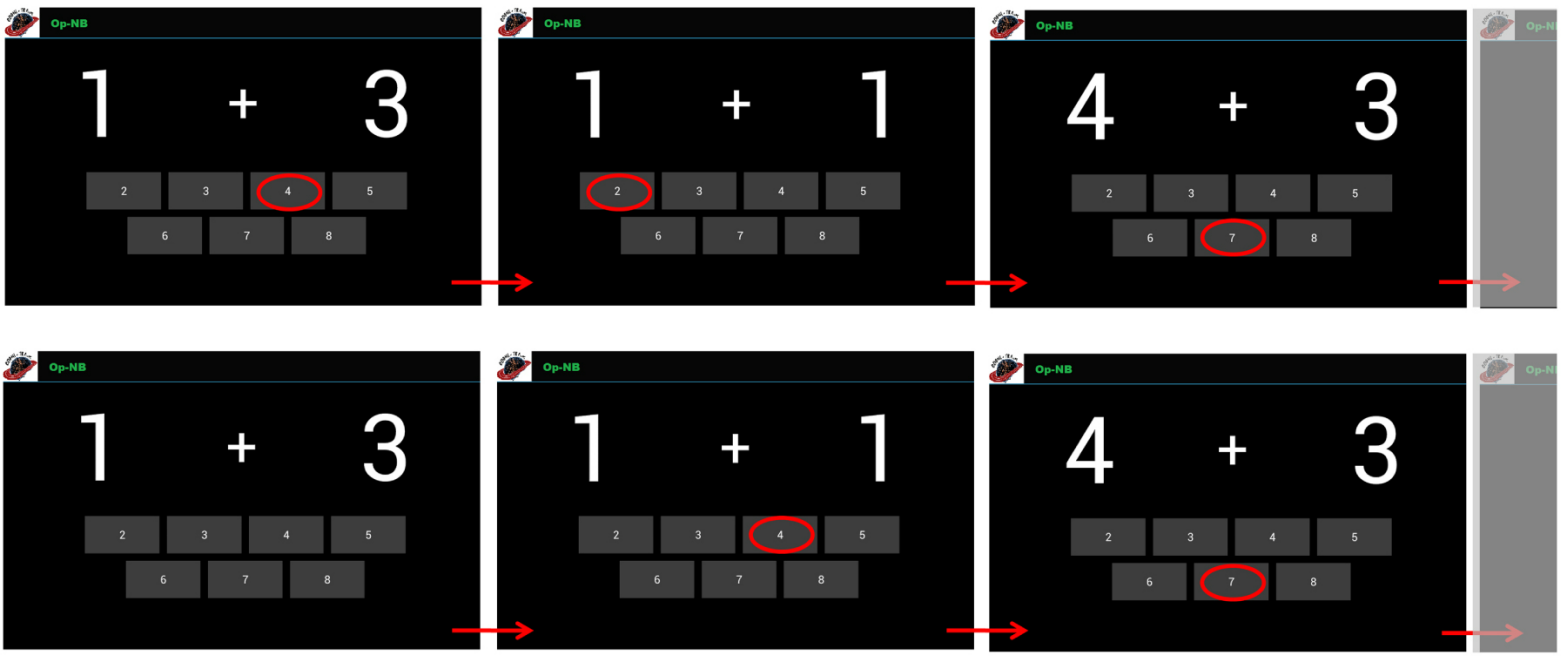
Examples of the operation N-back (Op-NB) exercises. The upper panel represents the operation 0-back (N=0) level of difficulty, whereas operation 1-back (N=1) is shown in the bottom panel. All the digits from 1 to 9 can appear on the screen and, consequently the sums range from 2 to 18. However, it is possible to reduce the difficulty of the task by limiting the digit span. Besides digit span, other task-specific parameters have to be taken into account such as the value of N (minimum 0; maximum not defined) and the rate of stimulus presentation. In Op-NB, adaptive working load algorithms operate only on N and rate of stimulus presentation.

For this exercise type (Op-NB), a pair of numbers are presented on the screen (eg, 1+4) and if N=0 (Operation 0-back), then the patients have to quickly touch the button corresponding to their correct sum. After each answer a new random stimulus (ie, a pair of numbers) appears. However, if N=1 (or higher), patients have to watch the pair of numbers, memorize them, and answer the correct result deferred by one (or more) new pairs. Thus, patients have to touch the button corresponding to the sum of N stimuli ago (Figure 2).

##### Dual N-Back

**Figure 3:**
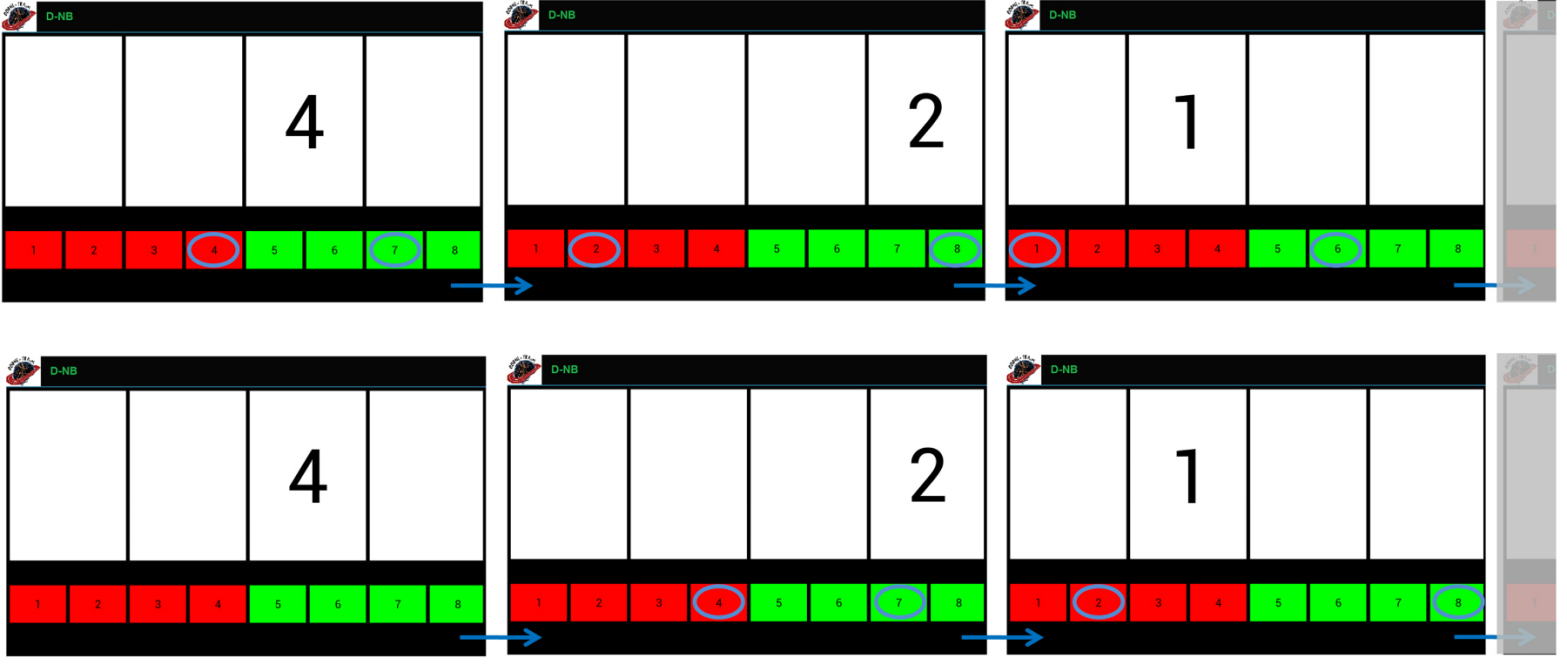
Examples of the dual N-back (D-NB) exercise. The upper panel shows the D-NB difficulty level when N=0 ("Dual" 0-back), whereas the lower panel shows N=1 ("Dual" 1-back). Here, patients have to watch the stimulus, memorize it, and touch the buttons corresponding to the digit and cell deferred by one new stimulus. Task-specific parameters are N (minimum 0; maximum not defined), and the rate of stimuli presentation, both susceptible to modifications by the adaptive working load algorithms.

In the dual N-back (D-NB) exercise type, patients have to look at a stimulus consisting of a digit from 1 to 4 on the screen. The stimulus appears in one of 4 adjacent cells placed in a row. If N=0 (Dual 0-back), the patients are asked to touch, as quickly as possible, both the response button corresponding to the appeared digit and the button indicating the cell in which the digit appeared. The red buttons (1, 2, 3, and 4), indicating the digit, are on the lower left corner of the screen and should be touched with the left hand. The green buttons (5, 6, 7, and 8), indicating cell position (5 represents the left-most cell and 8 the right-most cell), are found on the lower right corner of the screen and should be touched with the right hand. After each answer a new stimulus randomly appears. However, as for Op-NB, patients have to defer the answer according to the N value (Figure 3).

There are a fixed maximum number of trials for the Op-NB and D-NB exercise types; automatically created from the starting record, this parameter remains the same and is not subjected to the adaptive working load algorithms. Another crucial parameter not modified by the adaptive working load algorithm is the threshold of correct answers (in percentage) under which a trial is considered “not valid”. This parameter is independently set for the different types of exercises.

#### General Setting Section

After the exercise is assigned, general information can be set. In the panel for general setting, it is possible to set the daily maximum time of training for each exercise type (expressed in minutes). Consequently, in CR interventions in which an adaptive working load algorithm is checked, the total number of trials executed by the patient is not predictable as it depends on presentation rate of the stimuli and is adapted following the patients’ performances.

Several other important temporal options can be selected in the general setting section including the total duration of the CR interventions (expressed in weeks) and the maximum number of days a week allowed for the training (expressed in days). These options are particularly crucial in determining the intensiveness of the training and the possibility to administer personalized treatment. Moreover, in this section, the administrator can switch ON the User-check, activating the USER-modality that presents a more user-friendly interface to the patient. If User-check is switched ON, at each next tablet restart, COGNI-TRAcK is automatically loaded in the USER-modality that gives direct access to the training exercises for the selected patient. This modality is implemented in order to limit patient-device interactions and access to other apps or to the general settings of the tablet. As well, this modality allows patients to be completely autonomous in performing the treatment with only little experience in using portable devices, thus making at-home, self-interventions possible. The administrator can also switch OFF the User-check, returning to the ADMIN-modality for all the procedures reserved for the team supervising the training program.

#### Database

All the information entered in COGNI-TRAcK is stored in a database structure managed by SQLite database management system. The database structure consists of the following 3 sections (1) "Patients" containing the personal data of the patients, (2) "Exercises and Treatments" containing the records specific of each exercise type, the information of assignment, the checks of the adaptive working load algorithms, and the length of the CR intervention, and (3) "Settings" containing general COGNI-TRAcK configuration features.

Tables are consistently correlated according to an entity-relationship model. Each table is identified by a minimal set of uniquely identifying attributes (primary key) which point to indexes in other tables, establishing the relationships.

#### Routine for Data Processing and Adaptive Working Load Algorithms

Raw data recorded by COGNI-TRAcK are made available for data processing. After each trial execution, raw data are saved into a text file stored in a dedicated folder automatically created during the installation procedure. Routines for data processing are applied to elaborate raw data after each trial, to calculate the percentage of total correct answers to the stimuli, and to match it with the percentage threshold used to consider a trial “valid” or “not valid”. When one or more consecutive trials are “valid” or “not valid”, adaptive working load algorithms define a new record with the parameters adapted for the next trial (see Working Memory Exercises section). For each new record, the adaptive algorithms automatically modify one or more task-specific parameters depending on the level of difficulty.

A dedicated button generates a report containing the processed data of every task executed by the selected subject. Besides the date and time of task execution, description of exercise type, and paradigm parameters, the report contains the number of correct, incorrect, or missed answers given and the difficulty level achieved by the subjects.

The raw data and/or results text file can be exported to an external storage device. Moreover, through an Internet connection, the files can be backed up on the server at the rehabilitation unit for remote monitoring by the medical team. The backup can occur after each trial if the connection is activated during the training, or all the files not yet backed up can be sent when the connection is next activated.

### Study Design

#### Overview

In order to validate disposability-to-use of COGNI-TRAcK, a pilot test on patients with MS was performed by setting a specific training program with adaptive working load algorithms. A survey on its usability, motivation to use it, and compliance to the COGNI TRAcK treatment was performed at the end of the CR intervention.

#### Patients

A group of 16 patients with MS (3 men and 13 women) was recruited from the AISM Rehabilitation Centre of Genoa (Italy). The following inclusion criteria were considered for the recruitment process (1) a diagnosis of MS clinically defined following the McDonald criteria [25], (2) the absence of relapsing in the last 3 months, and (3) a performance lower than −1 SDs from the mean of one of the tests included in the Rao's Brief Repeatable Battery of Neuropsychological Tests (BRB-N) [26]. The BRB-N is the most widely used instrument to assess cognitive functioning in patients with MS and it is shown to be reliable and sensitive to identify disturbances of cognitive domains in these patients [27]. Normative values and correction factors refer to the Italian validation of the BRB-N, published by Amato et al [28].

The mean age of the patients was 49.06 years (SD 9.10, range 33-67) and their mean education was 11.75 years (SD 3.41, range 8-18). Of the patients, 9 were affected by a relapsing-remitting form of MS and 7 by a progressive form. The mean value of the Expanded Disability Status Scale (EDSS) [29], a method of quantifying disability in MS, was 3.75 (SD 1.92, range 1-6.5), and the mean disease duration was 161.69 months (SD 109.56, range 20-374). All the recruited patients performed a score lower than −1.5 SD in at least 2 tests of the BRB-N (see Multimedia Appendix 5). All of the patients were familiar with the basic usage of electronic devices such as personal computers or portable devices (ie, mobile phones or tablets).

All the patients that participated in this study gave informed consent. The study was conducted in accordance with the Declaration of Helsinki (1964) [30].

#### Training Program

The participants performed an 8-week, home-based training program of working memory exercises, scheduled in 5 daily sessions per week (40 sessions total), and each session lasting 30 minutes. This training schedule was planned in accordance to the study conducted by Takeuchi and colleagues [16], where they showed that a similar program administered to healthy subjects not only improved cognitive performance but induced a related brain structure recovery [16]. However, rather than the subjects executing the training every day of the week and more than once daily, we limited the training to only 5 sessions per week and one session a day. This schedule was chosen in order to avoid inducing central fatigue; in fact, central fatigue is a very limiting symptom experienced and reported by 60-90% of patients with MS [31].

All 3 types of working memory exercises implemented in COGNI-TRAcK (Vs-WM, Op-NB, and D-NB) were presented during each training session for about 10 minutes each. The starting level (starting record) for each type of exercise was set equal for all patients and the difficulty level varied according to the adaptive working load algorithm. The following algorithm was adopted: for every trial detected as "valid" the level of the task increased by 1 and a new record was automatically created. If 3 trials in a row were detected as "not valid", the level of the task decreased by 1 and a new record was automatically created. Accordingly, after the first and the second trial detected as “not valid”, no new records were created and the last used was adopted for the next trial. A trial was considered "valid" if the percentage of total correct answers was ≥100% for Vs-WM, ≥80% for Op-NB, and to ≥75% for D-NB, thresholds set according to the training program validated by Takeuchi [16].

For all 3 types of exercises, the level of difficulty was varied first until the rate of stimuli presentation reached a threshold (1 stimulus per second for Vs-WM and 1 stimulus every 3 seconds for Op-NB and D-NB), and then by changing the other task-specific parameters (number of stimuli for Vs-WM and N for Op-NB and D-NB). In Op-NB and D-NB, the number of stimuli increased with increasing N using the following mathematical formula:

Number of stimuli=(N+1)×5

According to this formula, when N=2, the number of stimuli is 15. In order to make the CR intervention more comfortable, participants were allowed to execute the daily 30-minute sessions at their preferred time of day.

The recruited patients underwent a practice session during which a neuropsychologist provided an explanation about COGNI-TRAcK functions and use, and provided detailed working memory exercise instructions. The neuropsychologist did not provide the system to start the intervention until he/she was sure that the patient was independent.

#### Outcomes

After the 8-week training program, a questionnaire the on usability of the system, motivation to use it again, and compliance to the treatment with COGNI-TRAcK was administered to each patient (Textbox 1), similar to previous studies on web-based CR tools [32]. The first 5 yes/no questions (Q1-Q5) investigated whether the patients felt ready to use COGNI-TRAcK at home after the practice session, whether they found the training interesting and useful, and whether they were motivated to use it again. Questions 6-9 (Q6-Q9) were multiple choice and required a single response from 1 (low) to 4 (high), indicating the level of motivation, stress, boredom, and amusement perceived while executing the exercises. The 4 possible answers in the multiple choice questions were "High", "Medium", "Low", and "Not at all".

As well, the percentage of days of completed training out of the total number of day in the 8-week period was calculated to assess the adherence to treatment (100% corresponded to the 40 sessions). Only the patients completing ≥32 sessions (80%, 32/40) were included in the analysis.

**Questionnaire assessing the usability, motivation, and compliance (disposability-to-use) of COGNI TRAcK.**

Questions

- Yes/no questions
  - Q1: Were the instructions on COGNI-TRAcK use given in the practice session clear and easy to follow?
  - Q2: After the practice session, did you feel independent to use COGNI-TRAcK?
  - Q3: Did you find the exercises interesting to you?
  - Q4: Did you find the training useful to you?
  - Q5: Are you motivated to use COGNI-TRAcK again?

- Multiple choice questions
  - Q6: What level of motivation to well perform did you feel during the exercises execution?
  - Q7: What level of stress did you feel during the exercises execution?
  - Q8: What level of boredom did you feel during the exercises execution?
  - Q9: What level of amusement did you feel during the exercises execution?

## Results

All the patients included in the data analysis performed the minimum of 32 sessions, with a mean adherence of 84% (33.4/40).

**Figure 4:**
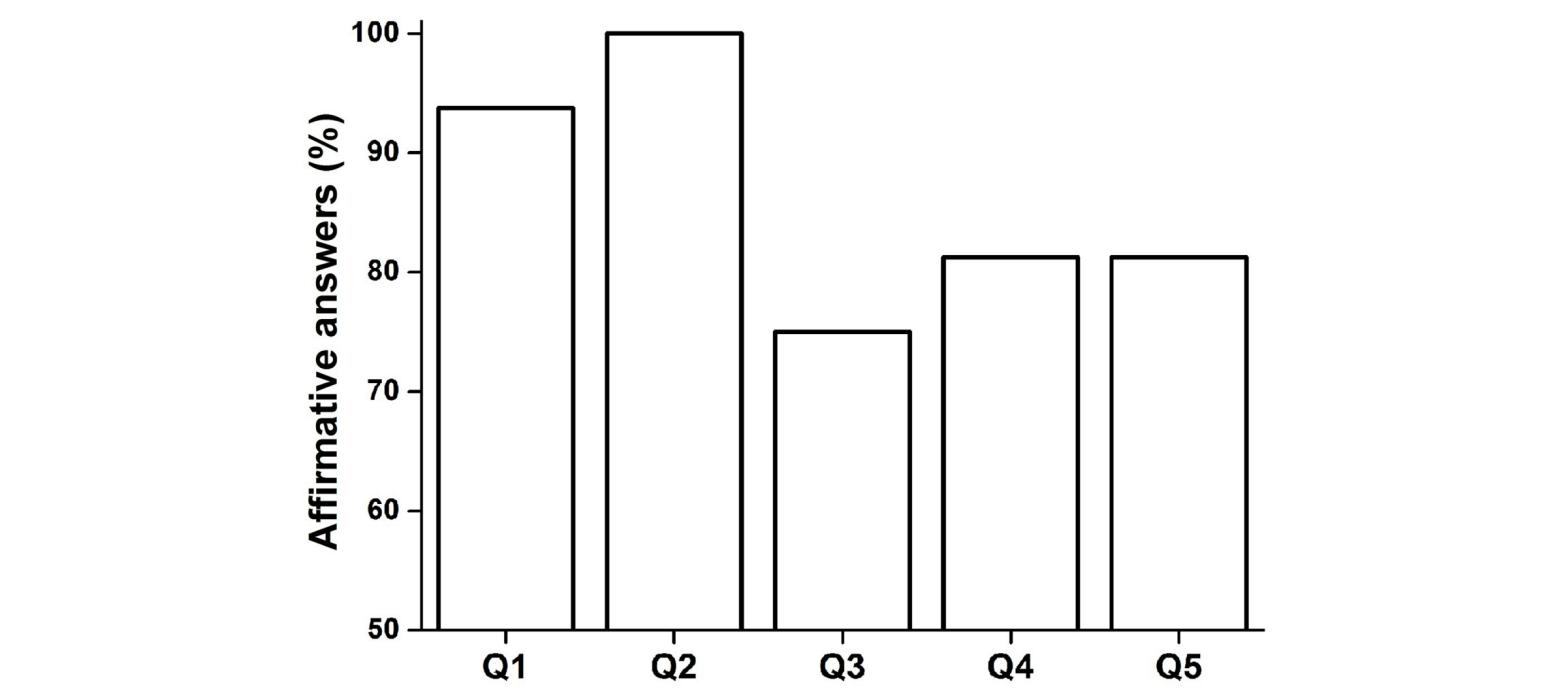
Results from the questionnaire on the usability of COGNI-TRAcK. The columns show the percentages obtained in the first 5 questions referring to usability and motivation.

**Figure 5:**
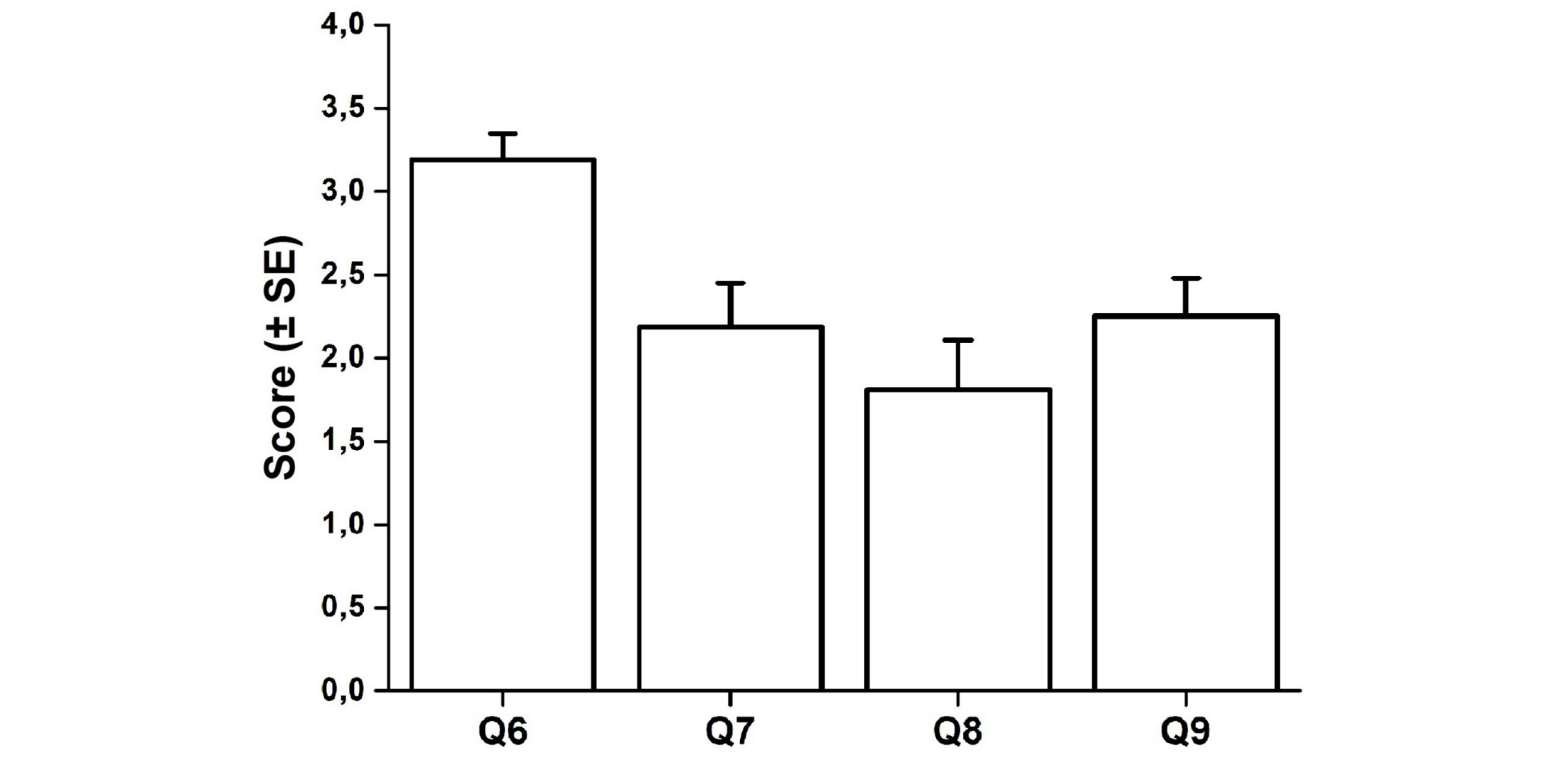
Results from the questionnaire on the motivation and compliance to use COGNI-TRAcK. The columns indicate the mean value obtained for compliance on a scale from 0 to 4.

Results from the questionnaire showed that 15 participants (94%, 15/16) understood the instructions given in the practice session (Q1), all participants (100%, 16/16) felt independent to use COGNI-TRAcK at home (Q2), 12 participants (75%, 12/16) found the exercises interesting (Q3), and 13 (81%, 13/16) also found the exercises useful for their clinical condition (Q4). After the 8-week training program, 13 patients (81%, 13/16) felt motivated to use COGNI-TRAcK again in the clinical practice (Q5) (Figure 4). Moreover, during the execution of the exercises, the patients felt highly motivated to perform well as the mean score for Q6 was 3.19 (SE 0.16). The patients experienced low levels of stress since the mean score for Q7 was 2.19 (SE 0.26) and were not bored as the mean score for Q8 was 1.81 (SE 0.30). As well, the mean score for Q9 demonstrating amusement was 2.25 (SE 0.23) (Figure 5).

## Discussion

### Principal Findings

Cognitive impairments often affect people with neurological disease [1,2], worsening their quality of life and reducing their occupational profile and social participation [3,4]. This is particularly constrictive for young patients, such as those with MS, which would like to remain independent as long as possible from the time of diagnosis, be active in the social context, and hope to maximize outpatient time fostering at home, self-administered interventions. In this context, CR would play a crucial role in reducing or maintaining the impairment stable, and facilitating the process of reintegration of these persons [19-21]. Thus, research focused on finding new ways to administer more usable CR interventions, to make them more effective, and to ensure a high adherence to the treatments is imperative. Rehabilitation researchers (ie, physicians, therapists, and bioengineers) should define and design new low-cost tools.

In this context, technology-based products such as mobile phones and tablets would meet these main requirements for future CR. COGNI-TRAcK is a tool meeting that meets these requirements since it is based on economic, accessible, and widely-used technological devices (mobile phones and tablets). COGNI-TRAcK presents an easy-to-use graphic user interface that allows for the self-administration and self-management of a home-based CR intervention and fulfills key factors such as adaptation and intensiveness of the working load that improve treatment effectiveness [5].

The present study assessed disposability-to-use of COGNI-TRAcK by investigating its usability, motivation for future use, and compliance to the treatment in patients with MS by an ad hoc questionnaire. Results show that this new system was very well received by patients with MS as deduced by the high adherence to the treatment. In fact, 84% of the total scheduled training sessions were completed by the patients, suggesting that this tool could be proposed for a CR intervention for patients with MS. As highlighted by the first 4 questions (Q1-Q4) in the opinion questionnaire, the app is highly usable. In fact, only one patient did not find the COGNI-TRAcK instructions given during the practice session clear and easy to follow (Q1). However, after probing deeper, it was revealed that the patient was referring to difficulties in correctly performing the exercises as instructed rather than difficulties with the general use of COGNI-TRAcK. Moreover, all patients felt independent to self-manage the training at home (Q2). In our opinion, this is a crucial result since previous studies in expectancy and usage of mobile technologies in the clinical environment revealed that a remarkable concern expressed by patients is that the use of mobile phones and tablets might be too complicated when it comes to health issues [24]. Of the subjects, 75% found the proposed exercises interesting (Q3) and >80% revealed that the training was useful (Q4). This important result shows that participants did not feel only the ludic aspects of COGNI-TRAcK but had an invested interest in performing the training and recognized the importance in stabilizing or ameliorating their personal cognitive performances. The question about motivation showed that the participants were highly stimulated to use COGNI-TRAcK again and adopt this tool in their clinical practice.

In general, compliance, investigated through the last part of the questionnaire (Q6-Q9), was found to be high. In fact, the participants felt highly motivated in performing the exercises (Q6), whilst the levels of perceived stress (Q7) and boredom (Q8) were low. However, low levels of amusement were experienced, suggesting that new expedients to increase interest and perceived amusement during exercise execution, such as graphical renovation, are required in order to enhance COGNI-TRAcK compliance.

### Conclusions

We demonstrated that COGNI-TRAcK, a tool for personalized cognitive intervention (self-administration), is highly usable, motivating, and well-accepted by patients with MS. We are now ready for a large-scale deployment of COGNI-TRAcK and are particularly interested in validating both its neurological aspects (eg, therapeutic effectiveness and effect on brain structure and function) and technological features such as Internet communication procedures for the data transfer to a central server.

## Multimedia Appendix 1

The main panel of COGNI-TRAcK consists of icons on the left to access the "Patients" and "Setting" sections. On the right, icons to access working memory exercises are displayed. The dashed icons represent new exercises that can be added to the app in future releases.

## Multimedia Appendix 2

Shown here is a view of COGNI-TRAcK used by the administrator where a new patient can be added and where the data of existing patients can be retrieved and managed.

## Multimedia Appendix 3

The "Treatment Assignment" section of COGNI-TRAcK shown here is used by the administrator to assign exercises for new trials of the rehabilitative treatment.

## Multimedia Appendix 4

The "Results" panel of COGNI-TRAcK is used by the administrator to see the results of already performed trials.

## Multimedia Appendix 5

The BRB-N performances obtained by the patients involved in the pilot testing. All the recruited subjects performed a score lower than -1.5 SD (red bolded colour) in at least 2 tests of the BRB-N (SRT-LTS: Selective Reminding Test-Long Term Storage, SRT-CLTR: Selective Reminding Test-Consistent Long Term Retrieval, SRT-D: Selective Reminding Test-Delayed, SDMT: Symbol Digit Modalities Test, PASAT-3: Paced Auditory Serial Addition Test-3 s, PASAT-2: Paced Auditory Serial Addition Test-2 s, SPART: SPAtial Recall Test, SPART-D: SPAtial Recall Test-Delayed, WLG: World List Generation).

## Abbreviations

BRB-N: Brief Repeatable Battery of Neuropsychological tests
COGNI-TRAcK: Cognitive Training Kit
CR: cognitive rehabilitation
D-NB: dual N-back exercise type
EDSS: Expanded Disability Status Scale
MS: multiple sclerosis
Op-NB: operation N-back exercise type
Vs-WM: visuospatial working memory exercise type
