# Supplementary figures and images for: A New App for At-Home Cognitive Training: Description and Pilot Testing on Patients with Multiple Sclerosis
Source: JMIR Mhealth Uhealth. 2015 Aug 31;3(3):e85. doi: 10.2196/mhealth.4269 (PMC4704979; doi:10.2196/mhealth.4269)

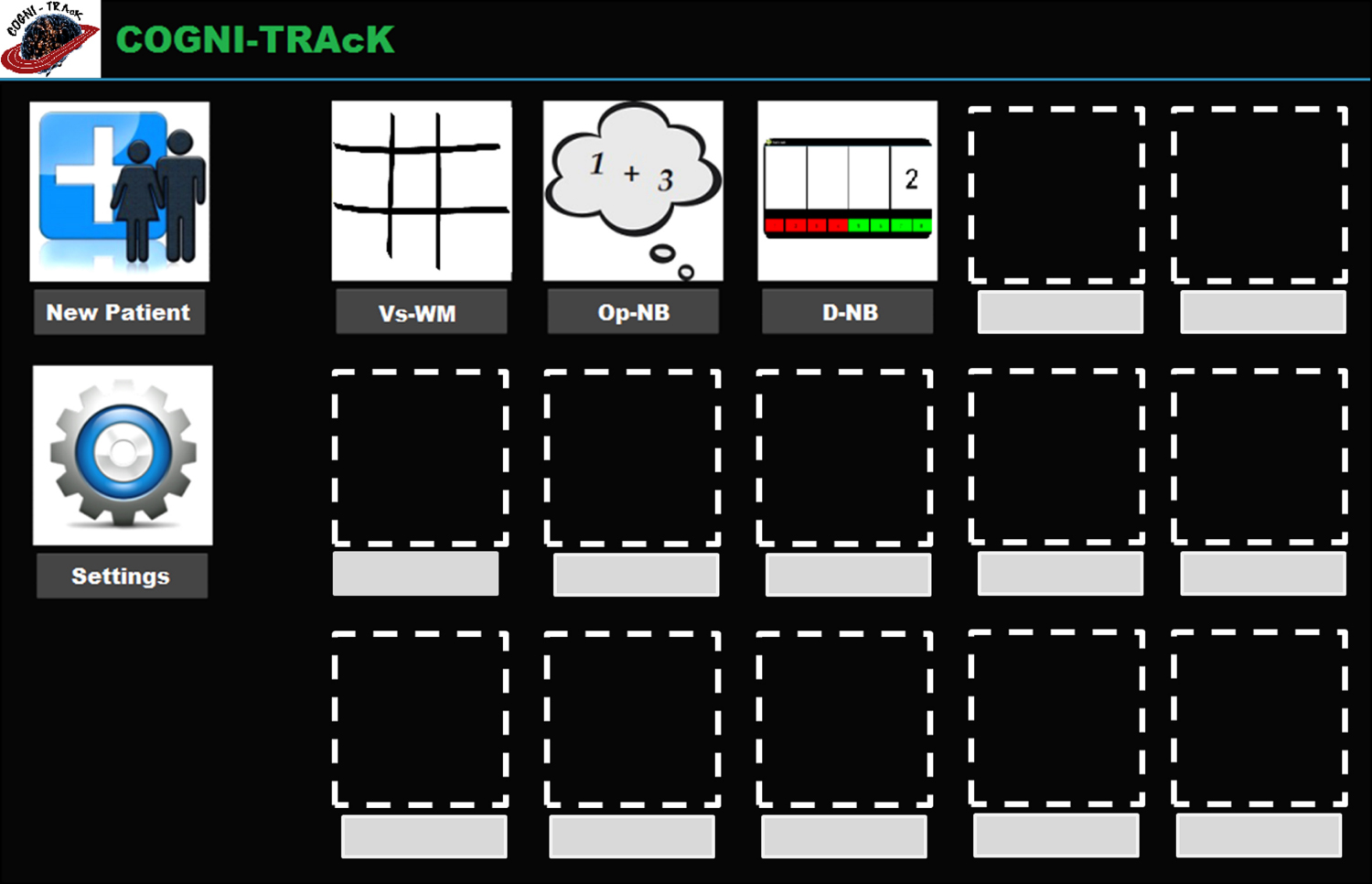

Supplement: Supplementary file 1 [file mhealth_v3i3e85_app1.jpg]

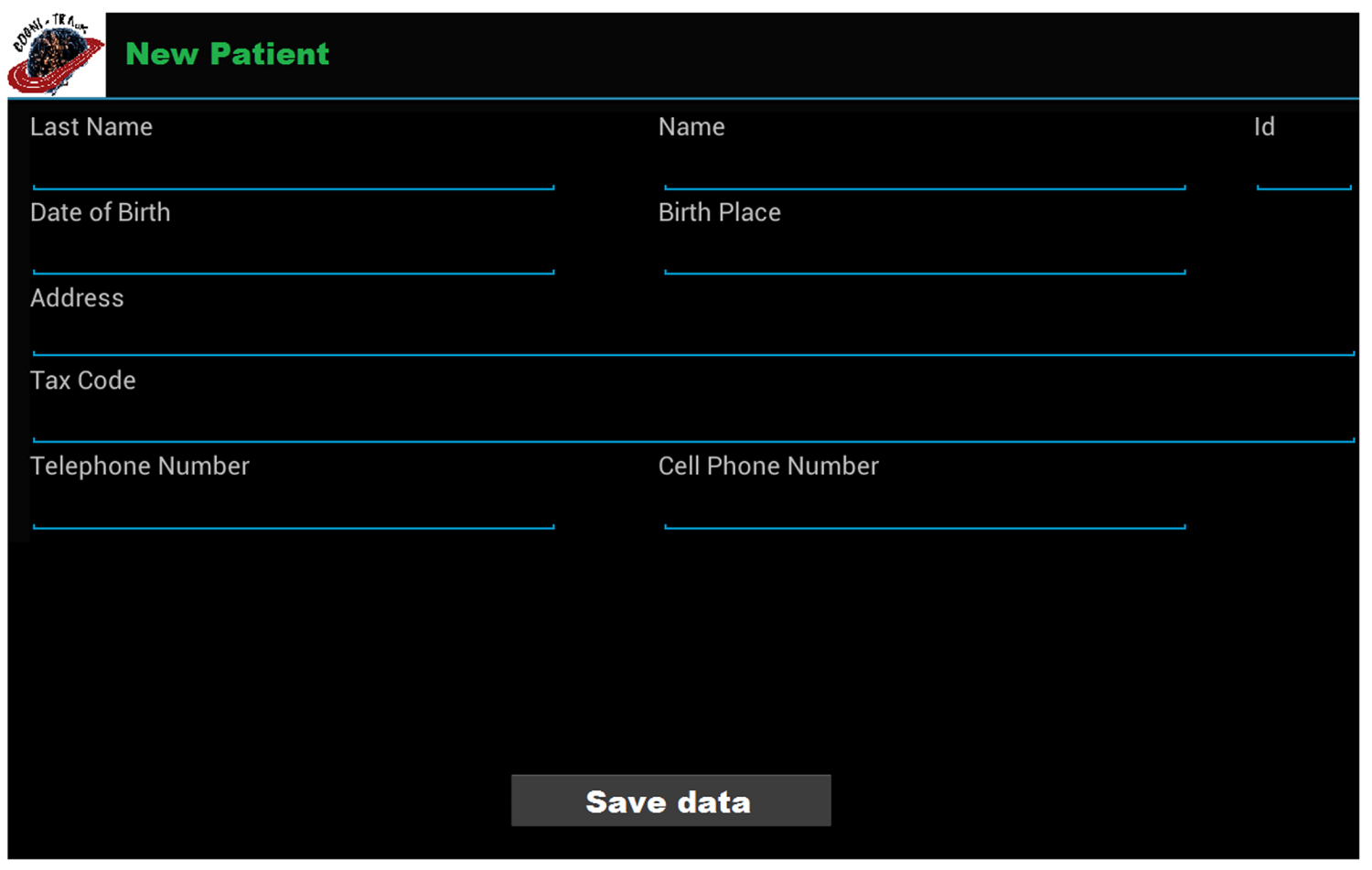

Supplement: Supplementary file 2 [file mhealth_v3i3e85_app2.jpg]

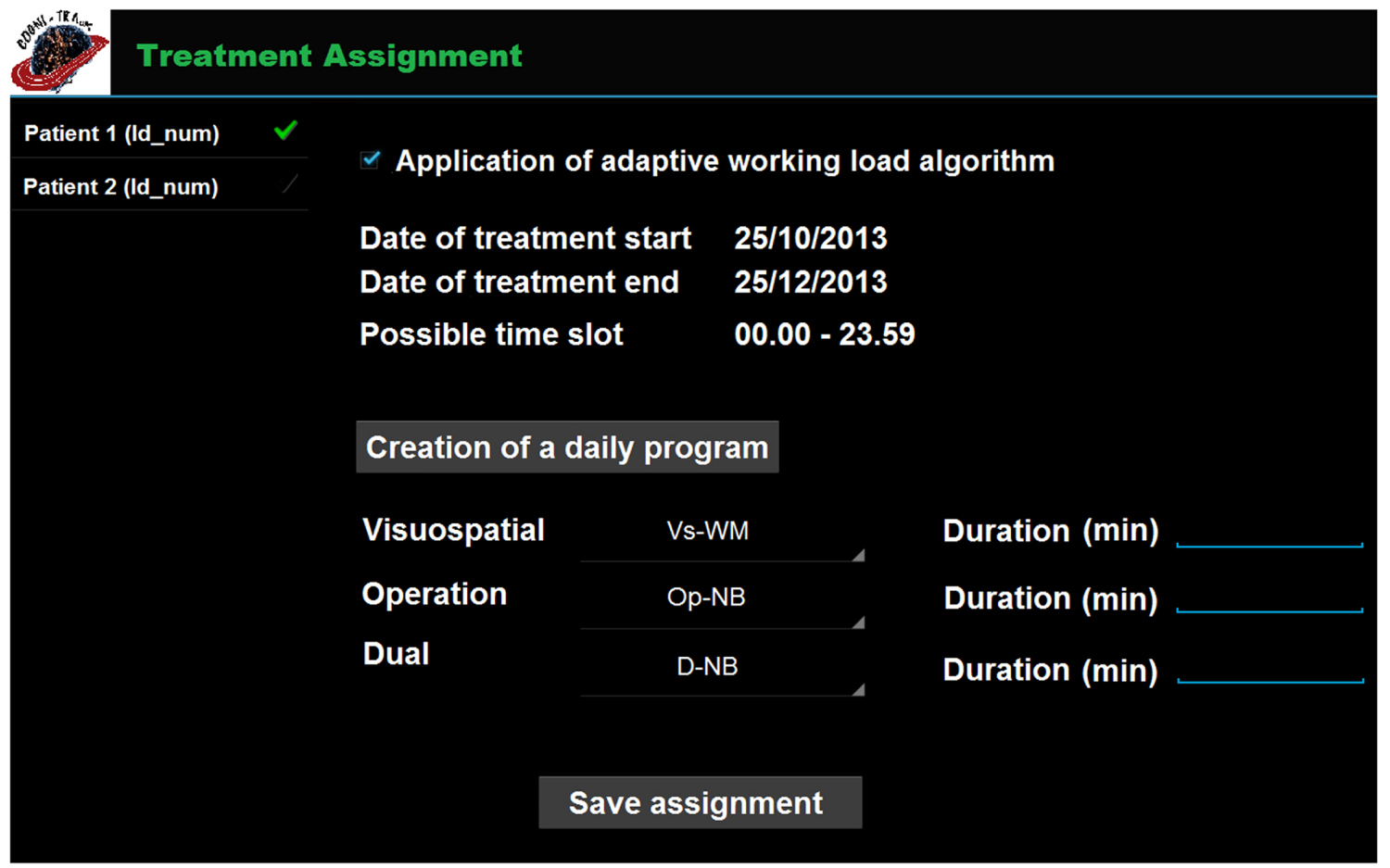

Supplement: Supplementary file 3 [file mhealth_v3i3e85_app3.jpg]

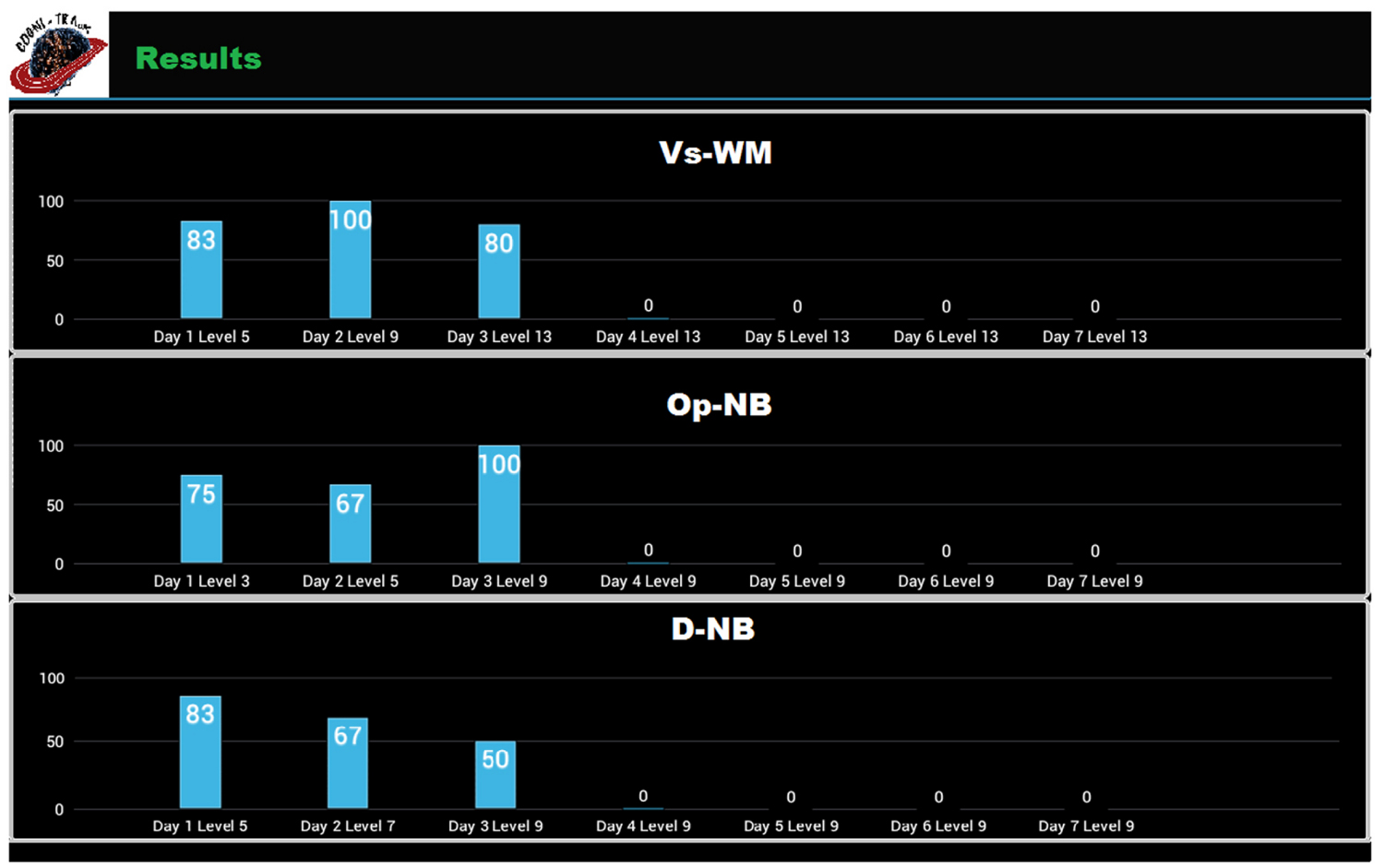

Supplement: Supplementary file 4 [file mhealth_v3i3e85_app4.jpg]

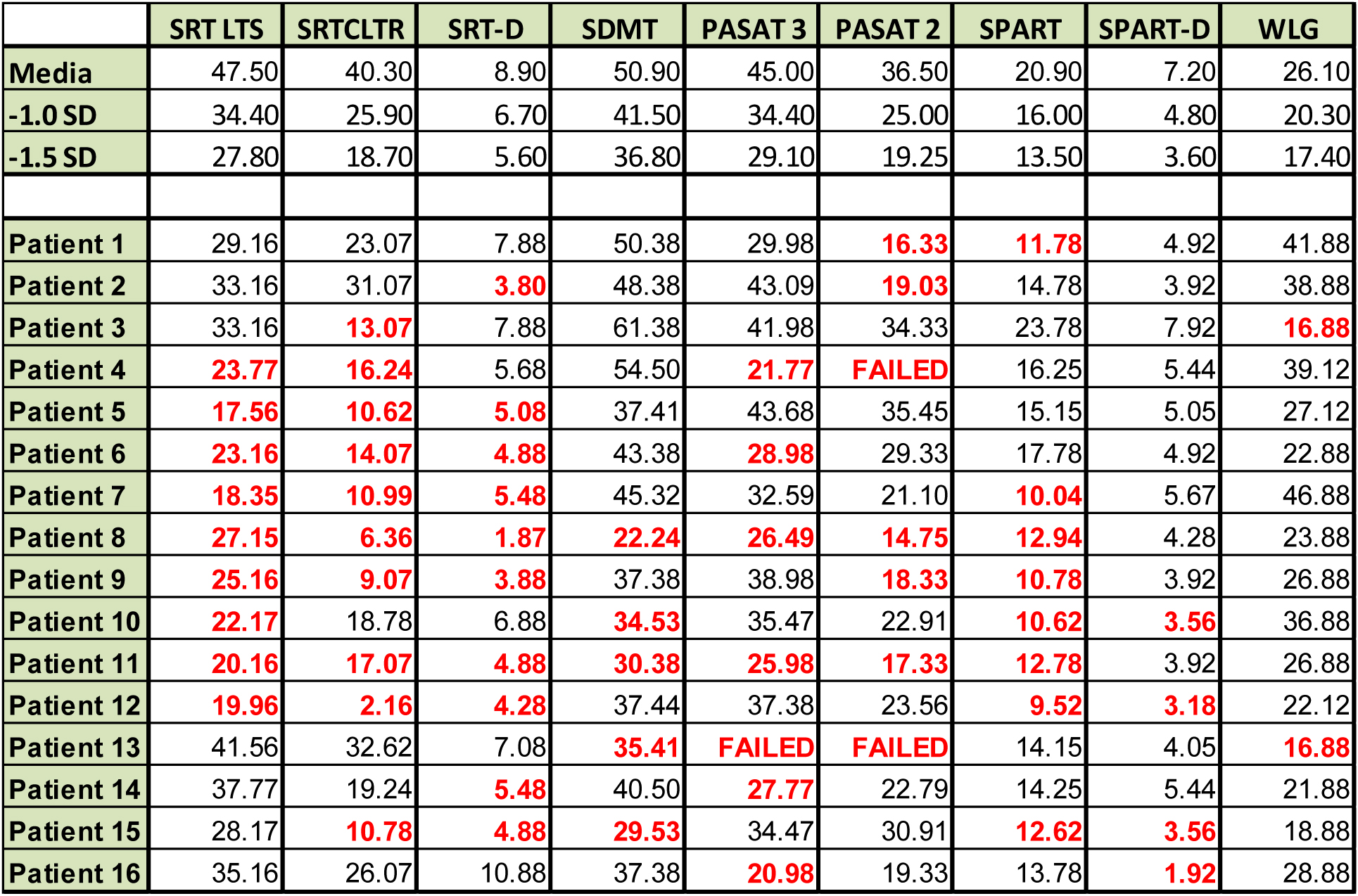

Supplement: Supplementary file 5 [file mhealth_v3i3e85_app5.jpg]
